# The Application of Additive Composites Technologies for Clamping and Manipulation Devices in the Production Process

**DOI:** 10.3390/ma16103624

**Published:** 2023-05-09

**Authors:** Richard Joch, Michal Šajgalík, Mário Drbúl, Jozef Holubják, Andrej Czán, Vladimír Bechný, Miroslav Matúš

**Affiliations:** Department of Machining and Manufacturing Technology, Faculty of Mechanical Engineering, University of Žilina, Univerzitná 1, 010 26 Žilina, Slovakia; michal.sajgalik@fstroj.uniza.sk (M.Š.); mario.drbul@fstroj.uniza.sk (M.D.); jozef.holubjak@fstroj.uniza.sk (J.H.); andrej.czan@fstroj.uniza.sk (A.C.); vladimir.bechny@fstroj.uniza.sk (V.B.);

**Keywords:** clamping device, composite, jaw, additive manufacturing, 3D print

## Abstract

Additive technologies have been widely adopted in various industries. The choice of additive technology and material directly affects the functionality of the manufactured components. The development of materials with better mechanical properties has led to a growing interest in replacing traditional metal components with those manufactured using additive technologies. The application of Onyx as a material comes into consideration, which contains short carbon fibers to increase the mechanical properties. This study aims to experimentally verify the viability of substituting metal gripping elements with nylon and composite materials. The design of the jaws was customized to meet the requirements of a three-jaw chuck of a CNC machining center. The evaluation process involved monitoring the functionality and deformation effects on the clamped PTFE polymer material. When the metal jaws were applied, significant deformation of the clamped material occurred, which varied with the clamping pressure. This deformation was evidenced by the formation of spreading cracks on the clamped material and permanent shape changes in the tested material. Conversely, nylon and composite jaws manufactured using additive technology demonstrated functionality across all tested clamping pressures, without causing permanent deformation of the clamped material, unlike the traditional metal jaws. The results of this study confirm the applicability of the Onyx material and provide practical evidence of the potential for reducing deformation caused by clamping mechanisms.

## 1. Introduction

Achieving precise machining is a complex process that depends on multiple factors. The aim is to ensure that the manufacturing conditions are suitable for achieving the intended design within the specified dimensional tolerances. As the demand for efficient and effective production increases, high-speed cutting (HSC) has emerged as a popular solution [1,2,3]. Although high-speed milling and grinding are widely accepted in serial production, HSC turning is still relatively uncommon due to the relatively short continuous cutting times and the lack of a safe and flexible clamping system capable of withstanding high-speed revolutions. The main reasons for this are the relatively short continuous cutting times and the lack of a safe and flexible clamping system that is capable of withstanding high-speed revolutions.

HSC turning requires high spindle rotation speeds, which can pose significant risks in the case of loss of clamping force or poor clamping mechanism condition. Therefore, it is crucial to select sufficiently high clamping force values to ensure the secure clamping of the workpiece. However, lower clamping forces can result in better tolerances [4]. Reducing friction may increase the risk of workpiece slippage, which can lead to collisions that can damage the machine and endanger personnel. Proper clamping of the workpiece during turning is essential for achieving the desired results. Currently, CNC machines are equipped with clamping devices that are directly controlled by the computer, such as pneumatic, hydraulic, and electromagnetic chucks. Of these, chucks are the most used mechanism for securing the workpiece during turning operations. Jaw chucks are the preferred clamping system due to their high flexibility regarding different clamping diameters. However, an important factor that must be considered is the distance between the machining site and the chuck jaws. As the distance increases, the stiffness decreases, resulting in worse quality parameters of the machined surface [5].

Another critical factor to consider is the material being machined. The clamping force must also compensate for strong bending moments and vibrations [6,7,8]. Studies have shown that factors such as clamping force, clamping length of the workpiece, and contact type between the workpiece and chuck jaws are primarily responsible for changes in stiffness [9]. High clamping force values can cause workpiece deformation, particularly in the production of plastic components.

Feng and Uhlmann have developed a computational model that takes into account both the stiffness of the chuck and workpiece to determine the dynamic clamping force of jaw chucks during high-speed turning [10]. It should be noted that hybrid CNC machines designed primarily for milling operations exhibit different kinematics during turning [11]. The precise calculation of the dynamic clamping force ensures a safe high-speed turning process and enables the full potential of jaw chucks to be utilized at high speeds. Legkiy also addressed the clamping of workpieces and created a prototype of clamping jaws without a counterweight [12]. However, this solution has a disadvantage: it requires the entire prototype to be manufactured, which is both time-consuming and expensive. Although contact between the chuck jaws and the clamped material and the resulting deformations are not desirable in the industry, a study was presented that uses deformations caused by clamping for forensic evidence between the workpiece and the machine tool used [13]. To reduce damage to the clamped material, adaptive metal jaws were designed based on CAE analysis. These jaws achieve complete contact with the jaw surface and reduce the resulting deformation on the clamped material [14].

These facts have prompted research into alternative clamping methods that can securely hold the machining material while minimizing the deformation and lowering the input costs. Furthermore, there is the aim to increase the modularity of the clamping devices.

To achieve secure clamping without damaging the shape of machined materials and reduce input costs, there are various potential solutions. One possibility is to use softer materials such as elastic silicone or nylon, which are also suitable for handling delicate objects such as plants [15,16]. Another approach is to apply composite materials with damping properties [17].

Additive manufacturing (AM) is a promising technology that enables the production of clamping jaws with virtually limitless possibilities by adjusting the material composition, processing conditions, and geometry of the object [18]. For example, Ferchow utilized laser-based powder bed fusion additive technology to produce integrated screws that serve as both a manipulation and clamping interface [19]. There are several AM methods available for producing a wide range of materials, including metals, polymers, polymer composites, and ceramics, which are classified into seven general categories according to EN ISO/ASTM 52900: 2022. AM is a digital manufacturing process that creates 3D objects directly from a CAD model by adding material layer-by-layer. In the past two decades, 3D printing has become an essential part of industrial innovation in various fields, particularly in rapid prototyping—thanks to its advantages [20]. Applying additive manufacturing can lead to quick production and modular design solutions.

A lattice structure is a type of cellular material that can achieve a variety of promising physical properties. Additive manufacturing (AM) has alleviated the difficulty of fabricating lattice structures with complex geometries. In the 3D printing process, several processing parameters have an impact on the mechanical and surface properties of the manufactured part. From FDM manufacturing, the presence of air gaps in the printed structure and stress concentration along the fiber affected the results by causing cracks [21]. Thus, the structure can absorb and dissipate higher energy by transforming it into internal energy through strain work [22]. The shape of the structure is important, which significantly affects the achieved mechanical properties. The results show that if the relative density of the auxetic cell core is appropriately chosen for a particular value of impact energy, then the sandwich panels with auxetic cores can have a higher level of energy absorption capability of up to 33% compared to rectangular and hexagonal sandwich panels [23]. In addition, the parameters of the additive manufacturing of the structure affect the resulting properties. The most important process parameter for inclined struts is the fan speed, but for horizontal struts, it is the height of the layer [24].

## 2. Materials and Methods

### 2.1. Composite Material for Additive Technology

Composite materials are created by combining at least two components at the macroscopic level, and their properties generally exceed those of their individual constituents [25]. These properties can include stiffness, strength, weight, and resistance to corrosion [26]. The matrix is the basic component of the composite and serves as a binder, while the reinforcing function is performed by fibers made of another material. Additive technology typically utilizes carbon fiber CBF, high-strength, high-temperature glass fiber HSHT, and continuous Kevlar fiber [27]. Depending on the length of these fibers, polymers can be reinforced with either long or short fibers, with composites reinforced with long fibers achieving better mechanical properties, where the tensile strength and Young’s modulus of the produced specimen with a carbon fiber content of 5 wt.% or 7.5 wt.% could increase by 22.5% and 30.5% [28]. As the number of fiber layers increases, the impact strength also increases. However, after a specific limit of the addition of fibrous layers, the increase in impact strength is limited [29].

For the clamping jaws, Onyx was chosen as the material. This technical thermoplastic was specifically developed for use on Markforged additive devices and is made of nylon PA6 filled with carbon fiber particles. Compared to traditional nylon, Onyx is about 3.5 times stronger. In addition, it has a higher hardness and a heat deflection temperature of 145 °C. The material properties for Onyx and carbon fiber reported by the manufacturer are listed in Table 1.

### 2.2. Design of the Clamping Jaws

**Figure 1:**
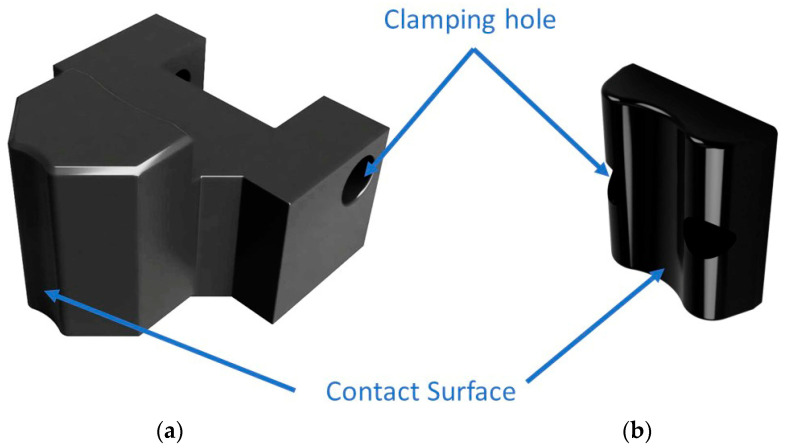
Design of the CAD model: (**a**) multi-purpose jaws; (**b**) outer jaws.

This design aims to create jaws with a suitable shape that can ensure proper clamping of shafts. The shape of the clamping jaws (Figure 1) is designed in CAD software Autodesk Inventor 2023 (Autodesk, Inc., San Francisco, CA, USA). Two types of prototypes were designed. The first type is multipurpose jaws (Figure 1a), which can clamp both the outer and inner diameters. The second type of jaws is designed as a separate outer clamping element (Figure 1b). When designing the outer jaws, the volume was significantly reduced to minimize the deformation zones. This design offers the advantage of smaller dimensions and higher stiffness. However, the disadvantage is that it can only clamp the component on its outer surfaces.

**Figure 2:**
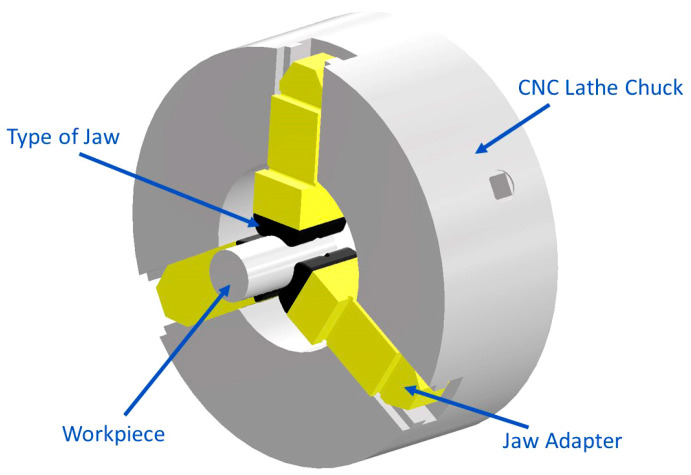
Visualization of the chuck assembly with three-point jaws.

The newly designed chuck jaws, manufactured using additive technology, are intended for use in a three-jaw chuck utilized in CNC machine tools (see Figure 2). Both types of the designed chuck jaws can be clamped onto the adapter from the rear and subsequently onto the chuck using screws. This solution ensures the required precision and stiffness of the jaw placement, as the metal jaw adapters incorporate guide grooves.

### 2.3. Additive Manufacturing of the Clamping Jaws

The mechanism of jaws was produced using composite additive manufacturing technology. The Markforged Two additive device (Markforged, Watertown, MA, USA) was utilized to manufacture the components. This device enables the reinforcement of objects with composite fibers via Continuous Filament Fabrication (CFF) technology. This additive technology is similar to FDM but includes a second print head for inserting fibers (such as Kevlar, carbon, or glass) into the component that is being fabricated. The combination of FFF and CFF technologies allows for the creation of composites that are suitable for even the most demanding applications, with a strength comparable to that of aluminum alloys [31]. The fracture toughness characteristic depends on the material’s elastic properties, which are highly dependent on the printer configuration in the case of 3D-printed composites [32,33]. In addition, the achieved strength properties are affected by the subsequent heat treatment [34]. To obtain the desired mechanical properties of the component, it is crucial to understand the impact of FFF technology parameters on the material properties. Parameters such as layer height, nozzle diameter, grid angle, or fill pattern significantly impact the mechanical properties achieved by functional components [35,36,37,38,39]. The products are significantly lightweight due to the internal fill and material density. This low-cost alternative to CNC machining minimizes waste and reduces operating and technological costs during manufacturing.

**Figure 3:**
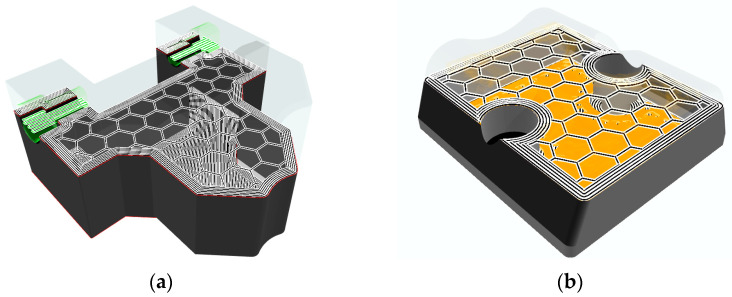
Internal structure of the jaws: (**a**) structure of the multipurpose jaws; (**b**) structure of the outer jaws.

The density of the infill and the shape of the material’s infill patterns in the form of hexagons, triangles, and rectangles affect the resulting strength of the material. Increasing the infill by 30% to 50% and applying the hexagonal infill will increase the energy absorption values [40]. The production of multipurpose jaws (Figure 3a) involved the use of an internal hexagonal structure with a density of 27%. This percentage was calculated and recommended by the software of the additive device. The height of the bottom and top layers was set to 0.4 mm, and the wall thickness to 2.4 mm. The entire additive manufacturing process took 22 h. To produce the outer jaws, the internal structure was oriented perpendicular to the machined surface (Figure 3b). Smaller dimensions allowed for an increase in the internal structure of up to 55%. To increase the stiffness of the jaws, two layers of carbon fiber were added as reinforcement (depicted in orange in Figure 3b), with layer thicknesses of 0.5 mm and 1.25 mm. The volume of onyx for one piece was 7.49 cm^3^ and the carbon fiber represented 0.76 cm^3^. The height of the bottom and top layers was set to 1 mm, and the wall thickness to 1.6 mm. The total manufacturing time of the jaws was 4.5 h. The placement of the model in additive manufacturing depended on the shape of the jaws. The goal was to achieve the greatest possible surface contact of the model with the printing table. Another factor was the elimination of print supports.

**Figure 4:**
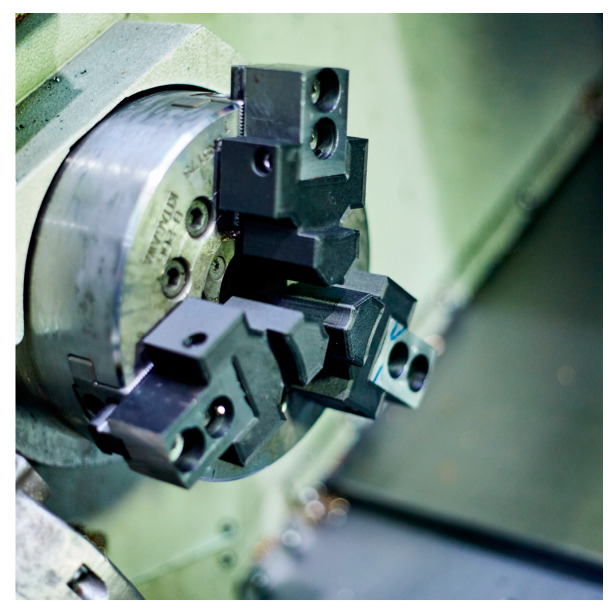
Jaws mounted on the chuck of the CNC machine.

The jaws were mounted on the chuck of the CNC machine (Figure 4). Both types of designed jaws underwent subsequent modification of their contact surfaces after the additive manufacturing process. The purpose of this modification was to ensure the roundness of clamping, thereby eliminating the possibility of workpiece displacement during the machining process of the machined material.

### 2.4. Design of the Experiment

The suitability of the additive technology for clamping jaws can be observed from various perspectives. This study aims to provide practical experimental verification. The principle involves the gradual testing of different types of jaws and monitoring the clamping process. The investigation was conducted on 12 identical specimens to ensure result variability. Therefore, it was necessary to set the same clamping pressures that were suitable for clamping the selected material (Table 2). To facilitate the deformation comparison, two reference specimens labeled R1 and R2 were included, which were not clamped in any type of jaws.

To monitor the deformation of the machined material surface due to the clamping pressure, it was necessary to select a material that would represent the soft materials. For this reason, PTFE polymer was chosen. The material was clamped in the machine jaws under the appropriate pressure (Table 2). Subsequently, the measurement of the resulting deformation on the clamped material was carried out. The clamping pressure was measured directly on the CNC machine, which includes a pressure sensor with an accuracy of ±1%.

### 2.5. Surface Measurement

The experimental part of the study focused on two factors: surface characteristics and the impact of clamping pressure on the deformation of the machined material. The surface was assessed through visual inspection, surface roughness, and profile. To characterize the shape, the roundness of the used material and circular waviness were monitored. Comparisons were made between standard jaws and designed jaw types. The machined surface was measured using the Alicona InfiniteFocus confocal microscope (Alicona Imaging GmbH Dr., Raaba, Austria). Geometric tolerances of cylindricity were evaluated using the MCYY (minimum circumscribed cylinder) method, which is typically used for assessing external surfaces. The portal CMM measuring device Carl Zeiss Eclipse (Carl Zeiss Slovakia, Ltd., Bratislava, Slovakia) with a ruby touch probe with a 1.5 mm diameter was used for roundness measurement.

## 3. Results

The results presented herein were obtained through the analysis of the data obtained from the conducted experimental activities.

### 3.1. Surface Characteristics

**Figure 5:**
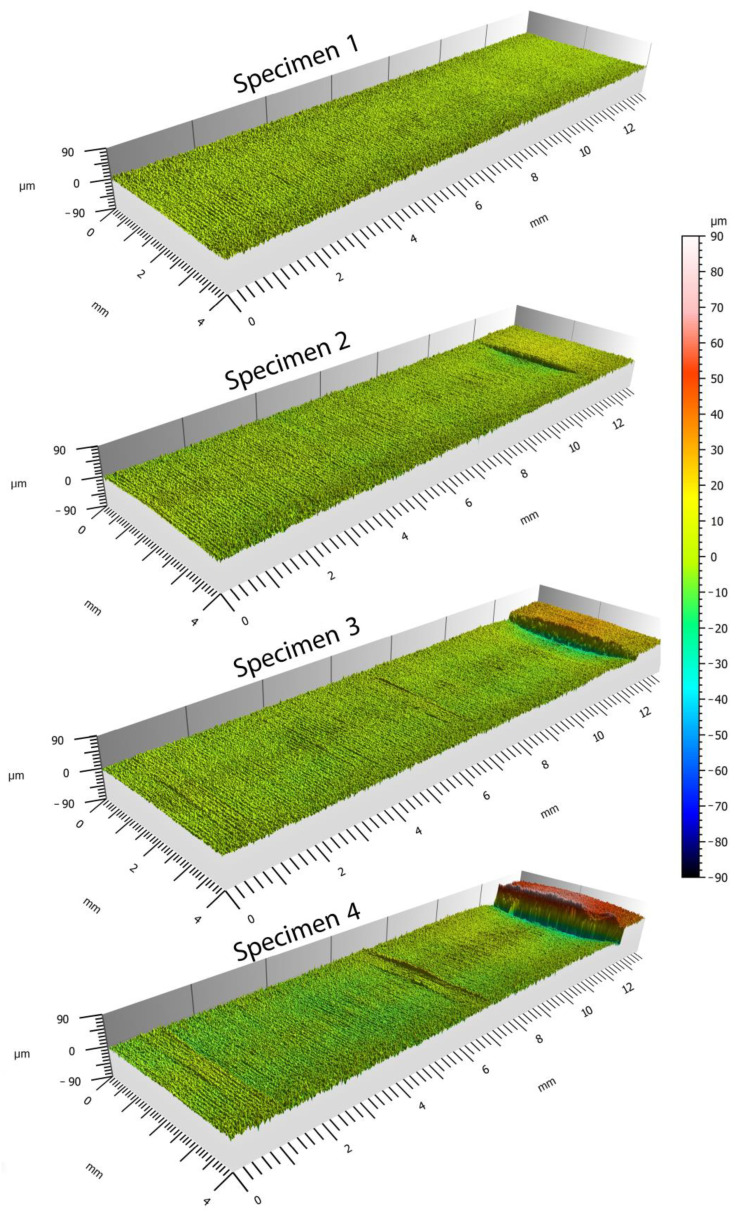
Visual inspection of specimens 1–4.

The specimens were visually inspected using a confocal microscope at 5× magnification (Figure 5). Significant surface deformation was observed in the specimens when standard metal jaws were used at a pressure of 1 MPa. As the clamping pressure increased, the surface deformation increased, leading to the formation of cracks at the point of contact between the specimen and the edge of the metal jaws, which were perpendicular to the workpiece axis. Specimen S1 did not show any visible surface damage. Specimen S2 exhibited traces of deformation at the point of contact with the metal jaw surface, resulting in a depression that did not tend to spread. Specimens S3 and S4 showed visible surface deformation due to clamping pressure.

**Figure 6:**
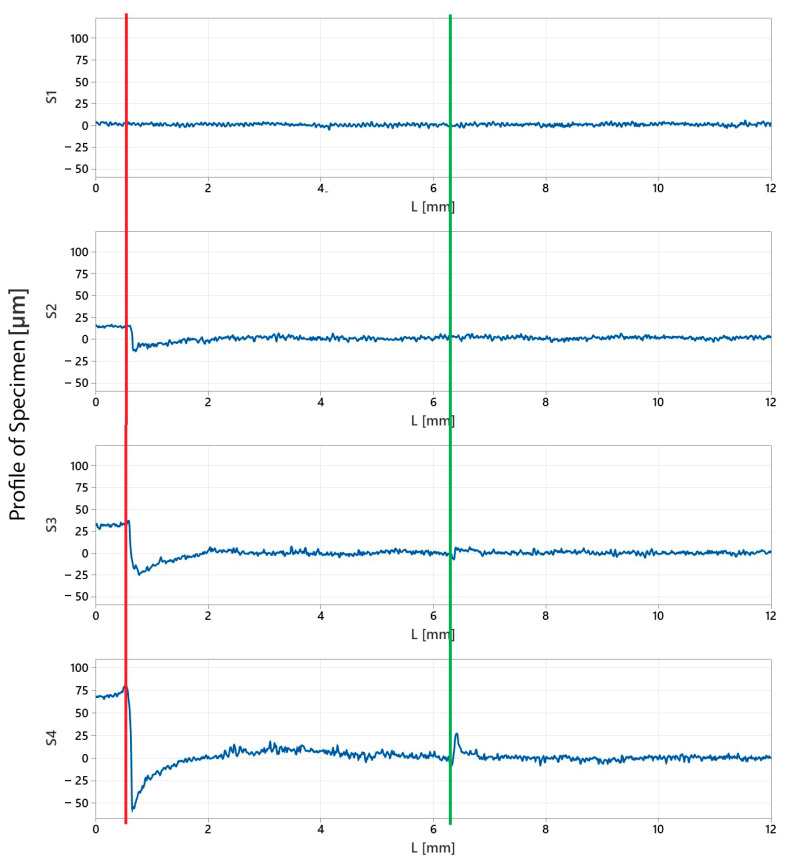
The profile of specimens S1, S2, S3, and S4 at the evaluated length L (red and green lines—places with specimen deformation).

Both visual inspection and 2D profile measurements revealed deformations after clamping with metal jaws. Figure 6 displays the measurement record showing the areas that came into contact with the edge of the metal jaws. The red line indicates the contact point of the side edge of the jaw, which was also visible during visual inspection. The resulting deformation is visible in the captured profiles. The metal jaws compressed the entire surface of the workpiece due to the clamping pressure. Compression reduced the radius of specimen S2 by 15 µm. Specimen S4 experienced the greatest compression of the material, with a radius compressed by 70 µm. The metal jaw had an edge perpendicular to the workpiece axis in its middle part, causing a similar but less significant deformation. The green line in Figure 6 shows the size of the deformation, which reached values of 5 µm for specimen S3 and 25 µm for specimen S4.

**Figure 7:**
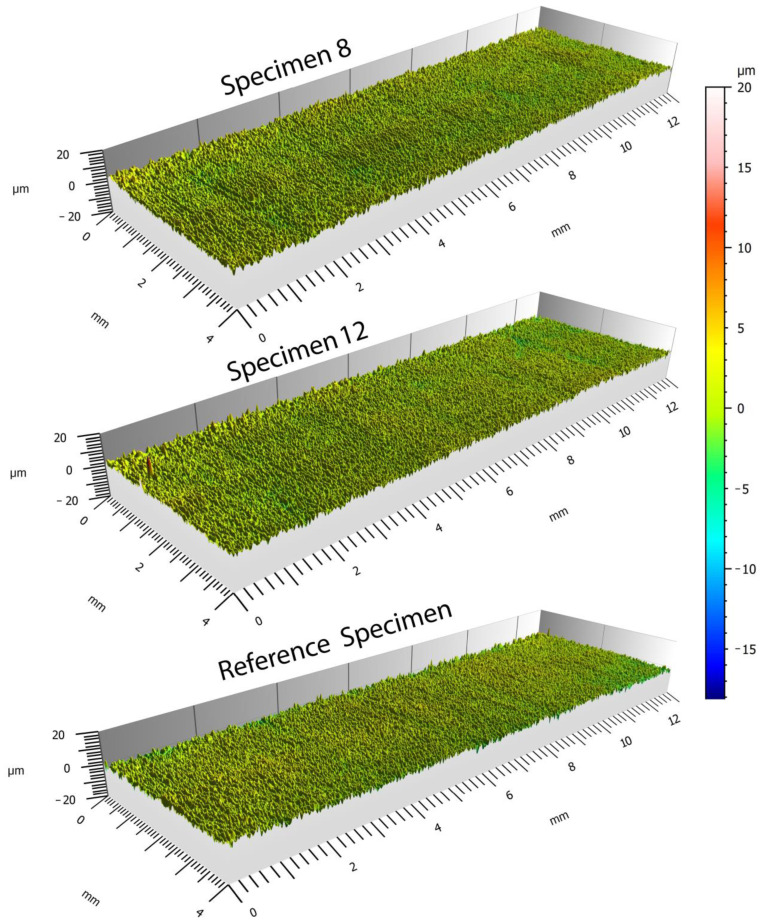
Visual inspection of specimens 8 and 12 compared to the reference specimen.

In comparing the reference specimens with those clamped using composite jaws at a maximum pressure of 3 MPa, there was no apparent damage observed (Figure 7). Therefore, it was necessary to focus on a parameter that could more accurately define the surface characteristics of the specimens after being clamped in the three-jaw chuck. The surface waviness parameter, Wz, is the sum of the highest peak height and the deepest valley depth of the profile. This parameter is derived from the primary profile, with the roughness wavelength suppressed.

**Figure 8:**
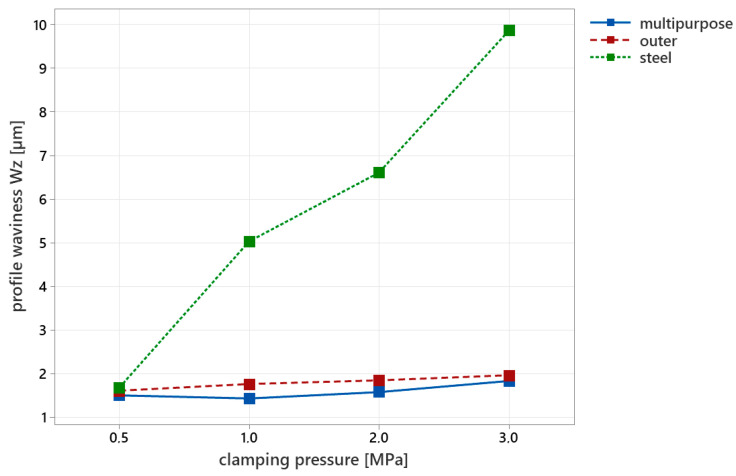
The surface waviness parameter Wz for specific types of used jaws.

The surface waviness measurement during filtration with λc 0.8 mm revealed a significant difference in the surface of the specimens depending on the type of jaws applied (Figure 8). Standard metal jaws produced the highest values of the waviness parameter, Wz. At the lowest pressure of 0.5 Mpa, the specimen waviness was 1.68 µm, but it increased to a Wz value of 9.86 µm at a clamping pressure of 3 Mpa. In contrast, when using multipurpose composite jaws, the maximum waviness values reached only 1.84 µm. Similarly, the outer jaws produced a maximum waviness of 1.96 µm at a clamping pressure of 3 Mpa. Overall, increasing the clamping pressure resulted in higher waviness parameters. The reference material, which was not subjected to clamping pressure, had a Wz parameter of 1.64 µm. Comparing the specimens used for different types of jaws, we found that the highest difference in waviness, compared to the reference material, occurred with the application of the metal clamping jaws.

### 3.2. Surface Shape

**Figure 9:**
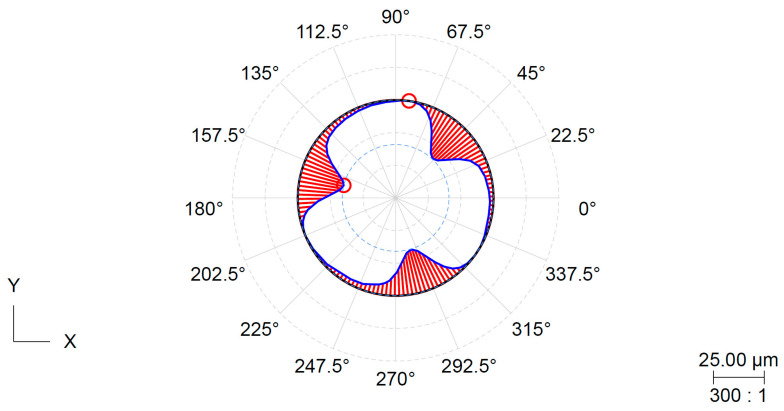
Record of roundness measurement for specimen S4 (metal jaws, pressure 3 MPa).

**Figure 10:**
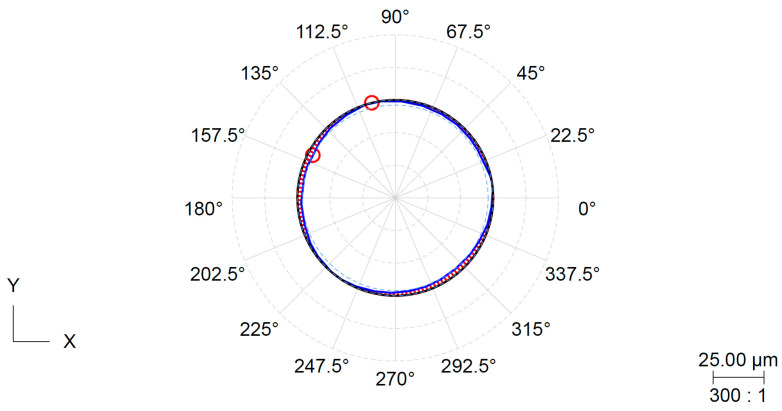
Record of roundness measurement for specimen S12 (outer jaws, pressure 3 MPa).

The roundness tolerances are classified under the form tolerance group. The surface being measured has filtered roughness and waviness components and is evaluated by comparing it with the ideal geometric shape. The roundness deviation is determined by the largest difference between these components. The minimum circumscribed circle (MCC) method was used, as per EN ISO 12181-1. The roundness parameter was measured for all the specimens, which had the same shape due to the applied clamping pressure. Figure 9 and Figure 10 depict that the shape character corresponds to the deformation derived from the three-jaw chuck.

**Figure 11:**
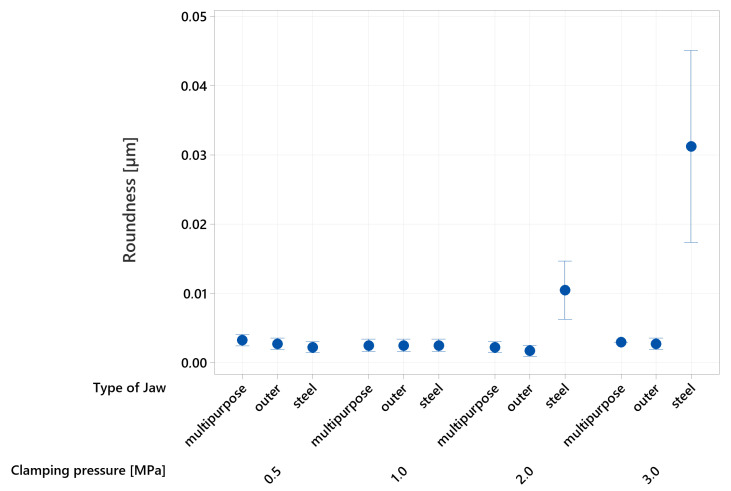
Roundness measurement of the specimens.

From the gained data presented in Figure 11, it is evident that the roundness of the specimens is influenced by the clamping pressure and the type of jaws utilized. Specifically, at a clamping pressure of 0.5 MPa, there are no significant differences in roundness values. However, at a pressure of 2 MPa, a significant increase in roundness value is observed when metal jaws are employed. The maximum average value of 0.03125 ± 0.01 µm is achieved when the specimens are clamped with metal jaws at a pressure of 3 MPa. This value is significantly different from the reference specimen, which had a roundness of 0.0048 ± 0.001 µm. No significant change in roundness values is observed when using either type of additive jaws.

**Figure 12:**
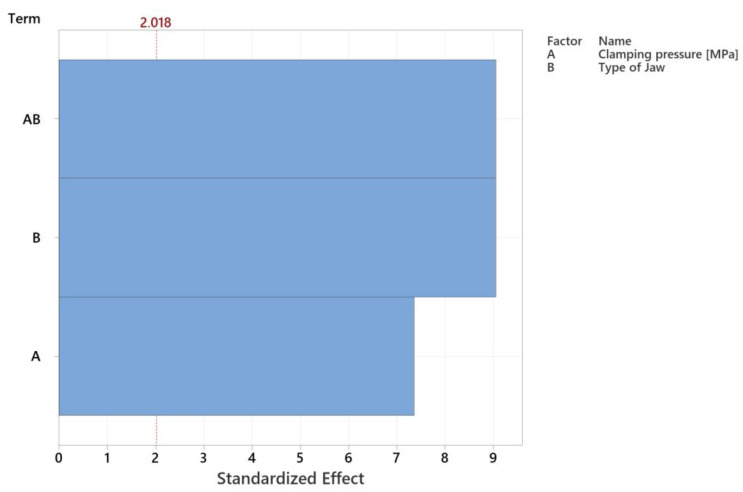
The effect of the selected parameters on the roundness of the specimens.

To understand the impact of individual parameters on the resulting roundness of the specimens, a Pareto chart of effects (Figure 12) was constructed with an accuracy of 85.06%. Our analysis revealed that both examined factors have a significant impact on the roundness of the specimens. Based on the effects, it is evident that the combination of clamping pressure and the type of jaws used, as well as the type of jaws alone, have the most significant influence on roundness. The size of the clamping pressure, while having a lower impact than the aforementioned factors, still plays a significant role in determining the roundness of the specimens.

**Figure 13:**
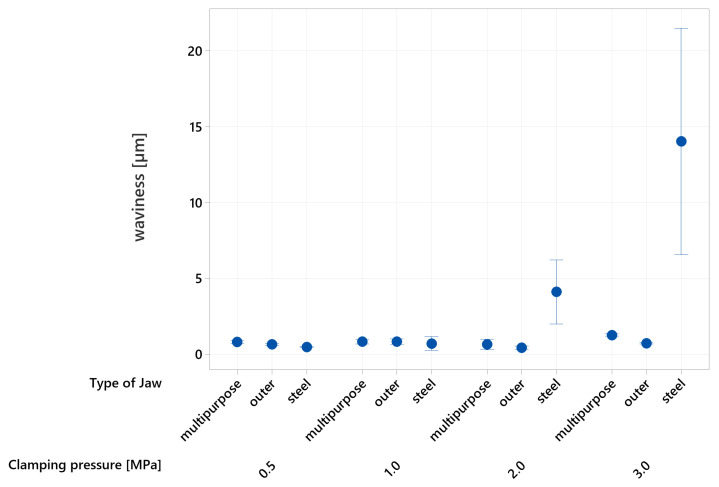
Circular waviness of the specimens.

Differences between the circular and profile waviness may arise from the contact surfaces of the jaws with the experimental specimens. This hypothesis is based on the fact that circular waviness measurement captures the whole circumference of the specimen, including regions beyond the contact areas of the jaws. The data collected indicate that the circular waviness of the specimens (Figure 13) exhibits a similar pattern to the roundness (Figure 11).

No significant differences in shape character were observed at the lowest clamping pressure. However, a notable difference was observed when the clamping pressure was increased to 2 MPa, resulting in an average circular waviness value of 4.12 µm. At the maximum examined clamping pressure of 3 MPa, the average circular waviness reached 14.06 µm. The circular waviness value for the unloaded reference specimen was 0.723 ± 0.006 µm, which corresponds to the equivalent circular waviness of specimens clamped by external composite jaws, where an average value of 0.738 µm was recorded. The precision of the influence of individual parameters on the resulting circular waviness was 82.35%, and their magnitude of influence was consistent with the effect on the roundness of the examined specimens.

## 4. Discussion

The conducted experimental activities indicate that the application of nylon and composite additive technology has the potential to replace the current materials. Instead of designing and producing the entire gripping mechanism [12], it is more economically advantageous to focus on changing the design of the shape of the jaws. [14]. The study demonstrated that the new jaws could withstand the same clamping pressures. Hence, we cannot claim that the gripping elements produced by additive technology are only suitable for delicate work [15,16]. Composite jaws also seem to be appropriate for HSC technologies that require the use of higher clamping pressures above 2 MPa.

By summarizing the findings, we can confidently state that there exists a significant difference between the conventional metal jaws and the novel nylon-composite jaws. Surface monitoring of the workpieces shows that deformation caused by the metal jaws already occurs at a clamping pressure of 1 MPa. This deformation can be classified into two types: deformation cracks and deformation across the entire contact surface. As the clamping pressure increases, the deformation cracks spread wider. The entire contact surface is subjected to surface pressure stress, leading to up to 70 µm alteration in specimen diameter at a pressure of 1 MPa. On the other hand, the new nylon and composite jaws remain without deformation on the clamped material even at a clamping pressure of 3 MPa. Deploying these jaws results in higher clamping pressure and better clamping conditions, with the possibility of utilizing the clamped material for machining and other manufacturing purposes. The jaw type also impacts the profile of contact between the jaws and the workpiece, with additive manufacturing-produced jaws exhibiting more favorable results. The new jaws showcase a 5× lower deformation effect compared to traditional metal jaws. Deformation is influenced by several factors, with both monitored parameters—clamping pressure and jaw type—significantly influencing the resulting deformations on the clamped material, with the type of jaws used having a higher impact. This effect unlocks the possibility of monitoring different materials and technologies suitable for this application (e.g., SLA, MJF, and SLS). Another crucial factor that may limit the use of nylon and composite materials is the temperature during the machining process. We assume that the temperature will determine the application limits, and therefore it is necessary to focus on its impact.

## 5. Conclusions

Clamping mechanisms are widely used across various industries, and constructing the mechanism itself is a common solution to achieve the desired properties. The current study demonstrates that, under specific conditions, nylon and composite gripping elements can replace previously used metal components. Experimental work reveals the following findings:

The designed jaws can be used with standard clamping devices without the need for special equipment.

- Components manufactured by composite additive manufacturing CFF are suitable for real conditions, and their application suitability is directly related to the material used and the working conditions;
- The designed nylon and composite jaws can withstand clamping pressures ranging from 0.5 MPa to 3 MPa;
- At a low clamping pressure of 0.5 MPa, all tested jaws achieve the same results, which means that the standard metal jaws can be used in an industrial environment;
- The type of jaws and clamping pressure directly affect the deformations of the clamped materials. However, nylon and composite jaws do not cause any deformations at the monitored clamping pressures;
- To ensure the continued usability of clamped materials, the application of metal jaws at clamping pressures higher than 1 MPa is appropriate only if the resulting deformation on the clamped part does not affect its functionality. It is crucial to consider the potential formation of spreading cracks and permanent changes in the diameter of the clamped material.

The results of the presented work and conducted experiments demonstrate that nylon-based composites achieve favorable results as clamping elements of the chuck. However, other parameters such as temperature stability, lifespan, and wear resistance need consideration for industrial application.

## Figures and Tables

**Table 1:** Mechanical properties of the material [30].

Parameter	Onyx	Carbon Fiber
Tensile modulus of elasticity [GPa]	2.4	60
Tensile strength [MPa]	37	800
Tensile stress at Break [%]	25	1.5
Heat deflection temp [°C]	145	105

**Table 2:** Clamping pressure parameters for the individual jaws.

Specimen Nu.	Type of Jaw	Clamping Pressure [MPa]
S1	Steel	0.5
S2		1
S3		2
S4		3
S5	Multipurpose	0.5
S6		1
S7		2
S8		3
S9	Outer	0.5
S10		1
S11		2
S12		3

## Data Availability

The data that support the findings of this study are available from the corresponding author (R.J.), upon reasonable request.
